# Enhancing physical activity and reducing symptoms of patients with osteoarthritis of the knee: a randomized controlled trial of the PrevOP-Psychological Adherence Program

**DOI:** 10.1186/s12891-023-06661-x

**Published:** 2023-07-04

**Authors:** Noemi Lorbeer, Nina Knoll, Jan Keller, Antonia Domke, Sally Di Maio, Gabriele Armbrecht, Hendrikje Börst, Peter Martus, Wolfgang Ertel, Ralf Schwarzer

**Affiliations:** 1 Department of Education and Psychology, Health Psychology Division, Freie Universität Berlin, Habelschwerdter Allee 45, Berlin, 14195 Germany; 2 Centre for Muscle- and Bone Research, Department of Radiology, Charité – Universitätsmedizin Berlin, Hindenburgdamm 30, Berlin, 12200 Germany; 3 Institute for Clinical Epidemiology and Applied Biometry, Universitätsklinikum Tübingen, Silcherstr. 5, Tübingen, 72076 Germany; 4 Department of Traumatology and Reconstructive Surgery, Charité – Universitätsmedizin Berlin, Hindenburgdamm 30, Berlin, 12200 Germany; 5 CARE-BEH Center for Applied Research on Health Behavior and Health, SWPS University, ul. Ostrowskiego 30b, Wrocław, 53-238 Poland

**Keywords:** Knee osteoarthritis, Osteoarthritis symptoms, Physical activity, Accelerometer, Planning, Action control, Health action process approach, RCT

## Abstract

**Background:**

This primary analysis evaluated the “PREVenting the impairment of primary Osteoarthritis by high-impact long-term Physical exercise regimen—Psychological Adherence Program” (PrevOP-PAP), designed to support patients with osteoarthritis of the knee (OAK) to engage in regular moderate-to-vigorous physical activity (MVPA) to reduce OAK symptoms (WOMAC scores). Theory-based on the health action process approach (HAPA), the intervention targeted volitional precursors of MVPA change: action and coping planning, maintenance and recovery self-efficacy, action control, and social network formation. We hypothesized that compared to an active control condition, increases in MVPA at the end of the 12-month intervention would translate into lower WOMAC scores at 24 months in the intervention condition.

**Methods:**

Participants with radiographically verified moderate OAK (*N* = 241; 62.66% female; *M(SD)* = 65.60(7.61) years) were randomly assigned to the intervention (51%) or the active control condition. WOMAC scores (24 months) were the primary -, accelerometer-assessed MVPA (12 months) the key secondary outcomes. The PrevOP-PAP was a 12-month intervention with computer-assisted face-to-face and phone-based sessions designed to increase HAPA-proposed volitional precursors of MVPA change (up to 24 months; secondary outcomes). Intent-to-treat analyses included multiple regression and manifest path models.

**Results:**

MVPA (12 months) did not mediate effects of the PrevOP-PAP on WOMAC scores (24 months). Compared to the active control condition, WOMAC scores (24 months) were lower in the intervention condition, but this effect did not remain stable in sensitivity analyses (*b*(*SE*) = -8.41(4.66), 95%-CI [-17.53; 0.71]). However, exploratory analyses revealed significantly stronger reductions in WOMAC-pain (24 months) in the intervention condition (*b*(*SE*) = -2.99(1.18), 95%-CI [-5.36; -0.63]). Groups did not differ in MVPA at 12 months (*b*(*SE*) = -3.78(3.42), 95%-CI [-10.80; 2.58]). Of the proposed precursors of MVPA change, action planning was higher in the intervention than in the control condition (24 months; *b*(*SE*) = 0.64(0.26), 95%-CI [0.14; 1.15]).

**Conclusions:**

Compared to an active control condition, the PrevOP-PAP did not produce reliable effects on WOMAC scores and none on preceding MVPA. Of the HAPA-proposed volitional precursors, only action planning was sustainably increased. Future interventions should use m-health applications to digitally support long-term changes in proposed volitional precursors of MVPA change.

**Trial registration:**

German Clinical Trials Register; https://drks.de/search/de/trial/DRKS00009677; also available at http://apps.who.int/trialsearch/; registration number: DRKS00009677; date of registration: 26/01/2016.

**Supplementary Information:**

The online version contains supplementary material available at 10.1186/s12891-023-06661-x.

## Background

Osteoarthritis of the knee (OAK) is a highly prevalent, progressive, age-related disease that causes pain and stiffness of the affected joint, causing reductions in individuals’ quality of life [1–4]. Recent estimations suggest that worldwide over 650 million persons over the age of 40 suffer from OAK [2]. Conservative treatment of OAK includes use of nonsteroidal anti-inflammatory drugs and different forms of physical activity (PA) [4, 5]. Guidelines recommend at least 150 min per week of moderate physical activity (MPA) or 75 min per week of vigorous physical activity (VPA), or a combination of both (MVPA), with added muscle strength, flexibility, and balance training [6]. This randomized controlled trial (RCT), which was part of the “PREVenting the impairment of primary Osteoarthritis by high-impact long-term Physical exercise regimen” (PrevOP) trial, was designed to enhance patients’ adherence to regular MVPA in order to reduce OAK symptoms (as measured by the Western Ontario and McMaster Universities Osteoarthritis Index, WOMAC [7]) [8].

Although patients with OAK report strong intentions to adapt their lifestyles to relieve severity or slow progression of OAK symptoms [9–11], high levels of pain and other barriers challenge uptake and maintenance of recommended PA levels [12, 13]. The health action process approach (HAPA; [14–16]), a psychological model of health behavior change, proposes key cognitions and self-regulatory strategies to conquer challenges that persons with OAK face when attempting to perform regular PA. In this theory, behavior change is subdivided in two phases: the motivational and volitional phases [14, 17]. Key predictors of the motivational phase include the following: Risk perception, i.e., perceived vulnerability and severity of suffering from progression of a disease if behavior is not changed [18]; outcome expectancies or pros and cons associated with the uptake and maintenance of PA [19]; finally, task self-efficacy or the belief that one is competent to change a behavior, e.g., regular PA [19]. These motivational precursors are then proposed to predict intention formation towards behavior change [14]. The subsequent volitional phase includes further predictors to bridge the intention-behavior gap including action planning to determine when, where, and how to perform the recommended behavior [14, 15, 20]; coping planning to identify barriers for regular PA and to prepare adequate coping strategies to deal with them [21–23]; and action control, where individuals monitor the progress and deviations from their PA goals and engage in regulatory efforts if their current behavior does not meet these goals [23, 24]. Two further volitional types of self-efficacy are proposed, i.e., maintenance self-efficacy and recovery self-efficacy. Maintenance self-efficacy addresses the belief that one is competent to maintain behavior change despite barriers. Recovery self-efficacy addresses the belief in one’s competence to resume the behavior following lapses or phases of inaction [14, 25].

The HAPA also considers contextual barriers (e.g., environmental conditions such as rainy weather) and facilitators to behavior change [15]. One such facilitating factor is the social network of the individual who wants to increase PA [26]. Network members may assist in target persons’ behavior change via providing support to become active or engaging in PA together with them and thus provide an added social benefit or strengthened commitment to the behavioral goal [27–31]. Intervention strategies that encourage the formation of collaborative implementation intentions (when, where, how, and how often are we going to be active together?) [32–34] were shown to help motivated persons to become more active together [34] or reduce being inactive in their daily lives [33].

There has been extensive research on PA in patients with OAK, however, only few intervention programs were theory-based [35–38]. Lack of theory-basis usually complicates the identification of active ingredients in interventions and also impedes the accumulation of evidence on intervention efficacy. Consequently, innovative theory-based interventions and intervention components are needed to facilitate the uptake and long-term maintenance of PA in patients with OAK. In this trial, we used the HAPA as a theoretical framework [14–16]. Although HAPA-based interventions have been designed and evaluated for different populations suffering from chronic diseases [15, 22, 39], HAPA-based interventions for persons with OAK are still rare. As a notable exception, the ENHANCE trial [38, 40] has evaluated a 12-week HAPA-based combined counselling and exercise intervention in patients awaiting hip and knee arthroplasty, with the aim to support patients’ uptake and maintenance of physical activity from pre- to up to 6 months post-surgery. Whereas both the intervention and usual care control groups showed improvements in PA and OAK symptoms in this time frame, authors did not find between-group differences. Moreover, initial between-group differences in proposed psychological mediators addressed by this intervention were not maintained at 6 months post-surgery [38]. This points out the need to extend the time of active intervention delivery by adding regular intervention boosters, a feature that was implemented in the present PrevOP Psychological Adherence Program (PrevOP-PAP) that delivered several intervention booster sessions over the span of 12 months [8].

Furthermore, most HAPA-based interventions so far have addressed contextual facilitators or barriers indirectly, for instance, as part of action or coping planning strategies where participants identify (alone or with others) good opportunities to act or barriers that keep them from implementing the planned behavior [15, 22, 32, 33, 39, 41]. Particularly for patients with OAK, a direct and systematic intervention-aided setup of contextual facilitators, such as social network formation, a novel intervention component of the PrevOP-PAP, appears promising, especially at later points of the intervention-assisted behavior-change process when intervention effects on self-regulation may start to decline [42]. To date, apart from general encouragement to seek social support if needed [38], an optional, but systematic social network formation intervention that also encourages the formation of collaborative implementation intentions to become active together with a chosen network member, has not been tested as part of HAPA-based intervention programs for patients with OAK.

In addition, current RCTs with patients with OAK mainly focus on shorter-term effects [38, 43], whereas longer term assessment periods are needed to understand causal mechanisms of complex interventions in the long run. Consequently, we aimed to test the effectiveness of HAPA-derived intervention strategies [22, 23, 32–34, 44–46], including social network formation, in the context of the adoption and maintenance of regular PA to reduce symptoms of OAK progression as part of the PrevOP-PAP using a research design with multiple assessments over an extended period of two years.

### Research question and hypotheses

In a population of individuals with moderate OAK, the present study addresses the following primary research question: Is there an indirect effect of a HAPA-based psychological intervention (PrevOP-PAP intervention), consisting of a motivational intervention and a volitional intervention including network formation, on participants’ OAK symptoms (WOMAC) via MVPA when compared to an active control condition receiving the motivational intervention only [8]? In the primary hypothesis we expected that compared to the PrevOP-PAP active control condition, participants receiving the PrevOP-PAP intervention would report decreased OAK symptoms (WOMAC) at 24 months following entry into the study and that this effect would be mediated by increased MVPA at the end of the active intervention phase (12 months post study entry). By investigating MVPA as a mediator, we thus aimed to elucidate the causal mechanism of a central active ingredient of the PrevOP-PAP intervention on the clinical outcome. In additional exploratory follow-up analyses, we further investigated intervention effects of the PrevOP-PAP intervention via MVPA at 12 months on different domains of OAK symptoms (WOMAC) at 24 months, i.e., WOMAC-functional limitations, WOMAC-pain, and WOMAC-stiffness. Secondary research questions and hypotheses addressed the predicted simple effects of the intervention on OAK symptoms (WOMAC) at 24 months, MVPA at 12 months, as well as HAPA-proposed precursors of change in MVPA at 24 months post study entry. Following a brief motivational intervention delivered to all participants, we assumed an overall increase in intention to engage in regular MVPA up to one week following the treatment. We further assumed differential long-term increases in HAPA-proposed volitional precursors of MVPA change that were directly addressed in the PrevOP-PAP intervention, that is higher 24-month levels of action planning and coping planning, maintenance self-efficacy and recovery self-efficacy, action control, and collaborative implementation intentions, as an indicator of social network formation, in participants of the PrevOP-PAP intervention condition, when compared to those of the PrevOP-PAP active control condition.

## Method

### Procedure, randomization, and design

The PrevOP-PAP was an unblinded randomized controlled trial embedded in a parallel group design with the PrevOP-PAP crossed within the same sample of the PrevOP-Main Medical Trial (PrevOP-MMT; preregistered at [47], see below and additional file (Additional Figure 1). Thus, PrevOP-PAP and PrevOP-MMT shared participants, inclusion and exclusion criteria, and the primary outcome of this study, OAK symptoms (WOMAC) [8]. Both PrevOP-PAP and PrevOP-MMT trials followed randomized, controlled, prospective, longitudinal designs.

**Fig. 1:**
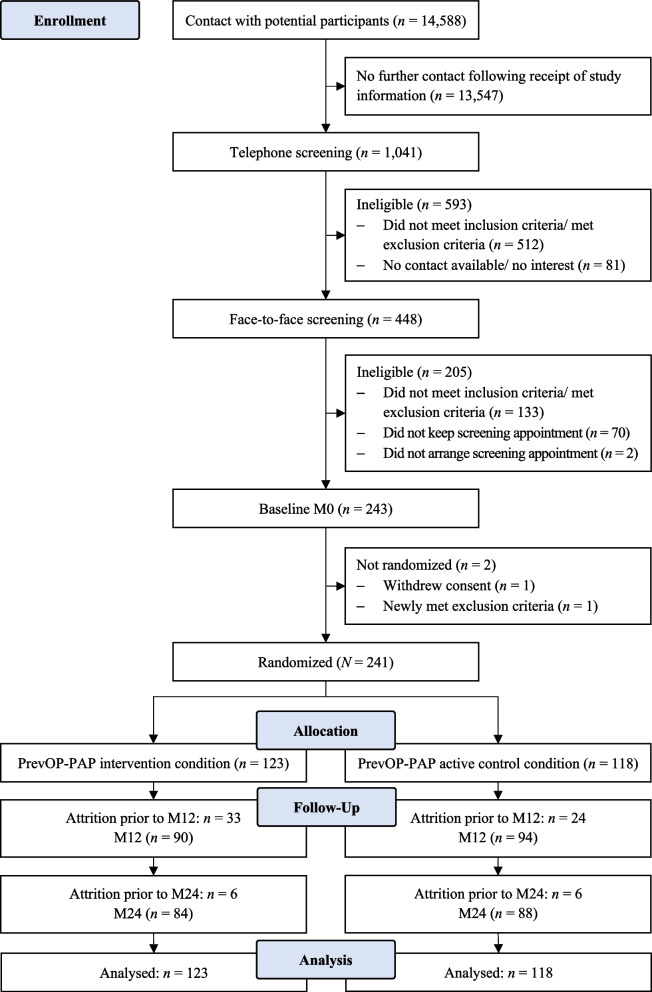
Consolidated Standards of Reporting Trials (CONSORT) diagram depicting participant flow through the study

The PrevOP-MMT tested a high-impact long-term physical exercise regimen with resistive vibration exercise (PrevOP-MMT high-impact exercise condition) against a low-impact long-term exercise regimen with walking exercise (PrevOP-MMT low-impact exercise condition) and an unstructured, non-monitored exercise control group (PrevOP-MMT active control condition). The PrevOP-MMT high-impact exercise condition and PrevOP-MMT low-impact exercise condition received structured and monitored training for 12 months, which was followed up by a home-based-mobility maintenance program (see trial registration [47]).

The PrevOP-PAP was crossed with the PrevOP-MMT (see below and additional file; Additional Figure 1), where a randomly assigned 51% of the total sample received the HAPA-based psychological intervention (PrevOP-PAP intervention, see below) and 49% served as the active control group (PrevOP-PAP active control condition, see below).

Individuals interested in study participation were informed about the study and screened for inclusion and exclusion criteria in an initial telephone interview and during a medical examination by PrevOP-MMT medical personnel at the study center at Charité – Universitätsmedizin Berlin. Prior to the medical examination, participants provided written informed consent.

Randomization of participants took place following baseline assessment (month “M”0) and was conducted at the Institute for Clinical Epidemiology and Applied Biometry at Tübingen University Medical Center, using computer-generated random numbers, stratified by sex. Participants were fully informed about randomization procedures and randomly allocated to one of a total of six intervention constellations (see additional file, Additional Figure 1): PrevOP-MMT high-impact exercise intervention (1) with the PrevOP-PAP intervention or (2) as part of the PrevOP-PAP active control condition; the PrevOP-MMT low-impact exercise intervention (3) with the PrevOP-PAP intervention or (4) as part of the PrevOP-PAP active control condition; or the PrevOP-MMT control condition (5) with the PrevOP-PAP intervention or (6) as part of the PrevOP-PAP active control condition. For the purpose of analysis, all participants allocated to the PrevOP-PAP intervention were collapsed in one study condition (51%) and all participants allocated to the PrevOP-PAP active control group were collapsed in another (49%), PrevOP-MMT condition allocation was controlled for (see below).

All participants received a brief motivational intervention following baseline assessment (M0) prior to randomization (see below and [8] for intervention content). In the PrevOP-PAP intervention condition, intervention periods lasted 53 weeks [8]. A main computer-assisted face-to-face intervention delivered by trained study personnel was conducted one week following baseline (M0) at the main study center. Four computer-assisted phone-based booster interventions took place at 3, 27, 50, and 52 weeks following M0 to ensure intensive intervention delivery at the start of PrevOP-PAP and booster sessions every six months following the respective data assessments. Additional paper–pencil activity calendar phases took place between week 1 and week 4, between week 25 and week 28, and between week 50 and week 53 following baseline (see below and [8] for intervention content). For computer-assisted phone-based interventions, participants were called at a location of their preference by trained study personnel. Paper–pencil activity calendars were completed daily by participants for three periods of four weeks each. Participants received travel cost reimbursement of EUR 5 per study center visit (assessment or intervention sessions).

In addition to six medical study visits with physical examinations and radiographic imaging as part of the PrevOP-MMT protocol (at baseline, 3, 6, 9, 12, and 24 months), data were assessed from all participants at baseline (M0), 6 months (month “M”6), 12 months (month “M”12), 18 months (month “M”18), and 24 months (month “M”24) via self-report measures and three one-week accelerometer assessments of daily PA (M0, M12, M24). Self-report measures were assessed via paper–pencil questionnaire booklets at the main study center (Charité – Universitätsmedizin Berlin, Germany; M0, M6; M12; M24) or at participants’ homes (M18) and returned directly to study personnel or mailed to the health pychology lab at Freie Universität Berlin. All data were collected between February 2016 (first assessment) and January 2021 (last assessment). The present report used data relevant for examining the primary research question with assessments at M0, M12, and M24 [8].

The ethics committee of the Charité – Universitätsmedizin Berlin approved this study (EA4/027/15). The present primary analysis report complies with CONSORT guidelines and TIDieR guidelines [48, 49].

### Power, recruitment, and inclusion

For the PrevOP-PAP, with an alpha level of 0.05 and a stability factor of 0.68 of the measure to assess the primary outcome (OAK symptoms as measured by the WOMAC [7]), a minimum sample size of* n* = 122 was determined which included 2 groups at 5 points in time up to the primary endpoint to detect a small effect (*f* = 0.1) of a within by between subjects factors interaction with a power of 0.95. To detect the proposed indirect effect of the PrevOP-PAP intervention condition on OAK symptoms (WOMAC) via MVPA with small-to-medium path coefficients (α = 0.26 and β = 0.26) and a power of 0.80 using bias-corrected bootstrapping with 2,000 resamples, a minimum sample size of *n* = 148 was determined [50].

With an expected drop-out rate of 20%, the required sample size increased to *n* = 153 or *n* = 185, respectively. For the PrevOP-MMT a sample size of *N* = 240 had been determined [8]. Reactive recruitment strategies were implemented throughout the greater Berlin (Germany) metropolitan area and included flyers, posters, social media, press-releases, and regional and national news-published interviews on OAK with calls for participation. Proactive recruitment strategies included mailings via local registration offices in the Berlin area (Germany) as well as recruitment of patients from an ambulatory clinic at the study center (Charité – Universitätsmedizin Berlin). Patients were recruited between February 2016 and November 2018.

Inclusion and exclusion criteria were mostly relevant for the medical PrevOP-MMT and are listed in the additional file (Additional Information 1) [8].

### Sample

Of *N* = 243 persons with OAK enrolled in the crossed PrevOP-PAP and PrevOP-MMT trials, *N* = 241 persons were randomly allocated to study conditions and *n* = 194 took part in at least 80% of the intervention sessions (i.e., at least 80% intervention fidelity, as assessed by the trained study staff) or were part of the control group. On average, participants of the PrevOP-PAP intervention condition attended 3.71 intervention sessions. At M24, data from *n* = 172 (71%) were available (see Fig. 1). The intent-to-treat sample was thus *N* = 241. For participant characteristics, see Table 1.

**Table 1:** Sample characteristics at baseline (M0)

	PrevOP-PAP intervention condition (*n* = 123)	PrevOP-PAP active control condition (*n* = 118)
Age in years^a^	65.46 (7.95)	65.75 (7.28)
Sex: Female^b^	77 (63)	74 (63)
Family status^b^		
Married	73 (60)	57 (48)
Committed relationship	15 (12)	14 (12)
Divorced	14 (12)^c^	29 (25)^c^
Single	11 (9)	16 (14)
Widowed	14 (12)	6 (5)
High school diploma^b^	73 (59)	62 (54)
University degree^b^	58 (48)	50 (42)
Employed^b^	47 (38)	40 (34)
Income (per month)^b^		
< €750	10 (9)	7 (6)
€750 to < €1250	20 (17)	25 (22)
€1,250 to < €2,000	33 (28)	34 (30)
> €2,000	53 (46)	47 (42)
Children^b^	101 (83)	86 (74)
Body mass index^a^	28.45 (4.27)	28.61 (5.45)
Kellgren-Lawrence grade		
Grade 2^b^	44 (36)	41 (35)
Grade 3^b^	79 (64)	77 (65)
Disease duration in years (knee osteoarthritis)^a^	11.41 (10.52)	11.64 (9.92)
Comorbidity^b^	98 (81)	101 (86)

229 ≤ *n* ≤ 241 participants due to missing values. Kellgren-Lawrence grade ranges from 0 (no knee osteoarthritis) to 4 (most severe knee osteoarthritis) [51]

^a^ Values are mean (standard deviation)

^b^ Values are *n* (valid %, rounded)

^c^ Frequencies with the subscript c differ at *p* < .05 between the PrevOP-PAP intervention condition and the PrevOP-PAP active control condition

### Masking

PrevOP-PAP intervention content could not be masked for study staff or participants. Study staff were aware of participants’ study group allocation at the beginning of the first intervention session of the PrevOP-PAP. Moreover, data analyses were conducted by N.L., N.K., and R.S. and were also unmasked.

### Intervention content

All intervention contents were delivered in German, derived from theory-based established intervention programs in primary and tertiary prevention settings [22, 23, 32–34, 44, 45], and adapted to patients with OAK in close collaboration with medical experts from the field. As part of a two-week piloting phase, ten patients with OAK of the outpatient clinic of Charité – Universitätsmedizin Berlin tested the intervention materials and provided feedback, which was subsequently used to further refine the intervention materials. For a more detailed description of all intervention components and materials used, see [8]. The intervention procedures were applied exactly as specified in the study protocol [8] with no modifications to the intervention during the course of the study. The intervention materials can be made available by the corresponding author upon request. Intervention contents were delivered by trained study staff (i.e., 2 to 3 Bachelor’s and Master’s students of psychology; employed as student research assistants in the trial) who were provided with training manuals and received training sessions on how to conduct the brief motivational intervention, the computer-assisted face-to-face, and the computer-assisted phone-based intervention sessions. PrevOP-PAP study researchers monitored the trained student research assistants’ intervention delivery throughout the study. Computer-assisted face-to-face intervention sessions were delivered at the main study center and computer-assisted phone-based interventions via phone. Intervention delivery was one-to-one, allowing for interaction between study staff and participant. All participants received the identical intervention with the optional network creation intervention as part of the third and fourth phone-based intervention (as described below). To prevent drop-out and maintain intervention delivery, participants were reminded via mail or phone prior to their appointment (face-to-face or phone-based intervention). If an appointment was not kept by patients, they were contacted via phone to reschedule the appointment.

#### Brief motivational intervention

Before randomization, all participants received a brief motivational intervention delivered by trained study staff that consisted of a brochure that participants were asked to read, followed by a brief quiz in form of a cross-word puzzle to test knowledge transfer. The brochure introduced participants to different intensities of PA, providing examples of joint-friendly MVPA and muscle-force strengthening exercises, and MVPA guidelines for persons with OAK [6, 52, 53]. It also addressed all motivational HAPA constructs [14, 15]: (1) risk of insufficient MVPA and evidence for consequences for OAK symptom progression (risk perception); (2) OAK-specific and generic benefits of increasing MVPA along with commonly perceived negative outcomes (outcome expectancies); and (3) use of self-instruction, recall of prior mastery experiences of increasing MVPA, calls for increasing MVPA in daily life, calls for thinking about role models for MVPA in participants’ social networks (PA-specific self-efficacy). In summary, intervention strategies used in the brochure comprised the following behavior change techniques (BCTs): goal setting (behavior) (1.1), social support (unspecified) (3.1), instruction on how to perform the behavior (4.1), information about health consequences (5.1), information about social and environmental consequences (5.3), information about emotional consequences (5.6), credible source (9.1), social reward (10.4), focus on past success (15.3), self-talk (15.4), and vicarious consequences (16.3) [8, 54] (see [8] for a more detailed description of the motivational intervention).

#### PrevOP-PAP intervention: computer-assisted face-to-face intervention

The computer-assisted face-to-face intervention, again delivered by trained study staff, consisted of an introductory section that reminded participants of the program’s goals, four ensuing sections focussing on outcome expectancies, self-efficacy, goal setting, planning, and a feedback section [8].

Outcome expectancies were addressed by providing participants with a calculated pros-cons difference score of outcomes of regular PA. Participants first indicated how much they agreed with five positive (e.g., less joint stiffness, good for overall health) and five negative (e.g., pain during specific activities, too time-consuming) outcome expectancy statements on 6-point Likert scales (not at all true to completely true). Then scores for pros, cons, and a benefit expectation difference were fed back to participants. In case of con scores being larger than pro scores, trained study staff reviewed concerns with participants and asked them to think of activities associated with less cons (BCT: pros and cons (9.2); [54]). Self-efficacy was addressed by asking for participants’ PA biographies and mastery experiences with PA throughout their life-span (BCTs: self-monitoring of behavior (2.3), identification of self as role model (13.1), identity associated with changed behavior (13.5); [54]). The goal setting section started with reminders of OAK-specific MVPA guidelines and joint-friendly activity examples [6, 52, 53]. Then, testimonials were provided that depicted a 61-year old man and a 68-year old woman describing their PA goal pursuits. Participants then recorded up to five of their own PA goals (i.e., type of activity and duration), including new activities and those that they already performed (BCT: goal setting (behavior) (1.1); [54]). Participants’ PA goals were reiterated one-by-one during the planning sections and participants were asked to create action plans for their goals. Plans should be phrased as “If/When…, then…” sentences with specific cue-situations (If/When) connected to the PA goal-activity (then). Participants then indicated on a 6-point scale (not at all true to completely true) their plan-execution self-efficacy, named a start date and were asked to copy their plans, as presented on the computer screen, into provided paper-pen activity calendars (see below). Subsequently, each action plan was shown to participants again and they were asked to generate a coping plan by identifying a potential barrier that may keep them from following through with their action plan (If/When-part of the coping plan) and specify how to manage this barrier (Then-part of the coping plan; e.g., by performing a different activity or choosing another time/place) (BCTs: action planning (1.4); coping planning (1.2) [54]). All plans were then shown to participants on summary screens. A feedback section ended the computer-assisted face-to-face intervention. The computer-assisted face-to-face intervention session lasted 60 min (see [8] for a more detailed description of this part of the intervention).

#### PrevOP-PAP intervention: computer-assisted phone-based intervention and activity calendars

Trained staff (i.e., trained Bachelor’s and Master’s students of psychology employed as students research assistants in the trial) followed a computer-based structured intervention [8], designed to boost participants’ planning, self-efficacy, and action control concerning regular PA and recorded participants’ responses in the program that provided the intervention contents produced by participants during the last session.

To increase maintenance self-efficacy and recovery self-efficacy, participants were first asked to review their PA-plan pursuit and indicate a success rate of implementing their PA-specific plan enactment in percent. To do so, participants used their completed activity calendars of the two weeks prior to the phone-based intervention. Participants were then asked to recall positive experiences with implementing their PA plans during the past two weeks. Following this, participants were given the opportunity to revise PA goals and associated action and coping plans or add new ones, up to a maximum of five. This was done in the same manner as in the computer-assisted face-to-face intervention with interventionists recording and reading out the intervention content to participants. If plans were kept, participants were asked to rate their plan-execution self-efficacy anew. At the end of this section, interventionists repeated each kept, altered, or new action plan aloud and asked participants to fill them into a new set of activity calendars. A summary print-out of all action and coping plans generated during the phone-based intervention session was also sent to participants’ homes.

Phone-based interventions 3 and 4 had an additional optional component of network creation, when participants were encouraged to identify a sports companion, contact them (phone-based intervention 3) and include these companions (i.e., their initials) into their action plans, creating collaborative implementation intentions (phone-based intervention 4) (BCTs: action planning (1.4), social support (practical) (3.2); [54]). If participants preferred to be active without a companion, these sections were skipped.

Computer-assisted phone-based interventions lasted between 20 and 60 min. At the end of each intervention session (face-to-face or phone-based) participants rated the quality of the session, were asked if they had any questions, were reminded of the next study appointment, were asked to use the self-regulatory strategies in their daily lives, and were thanked.

The final component of the PrevOP-PAP intervention were paper–pencil activity calendars to promote action control and maintenance self-efficacy as well as recovery self-efficacy using BCTs self-monitoring of behavior (2.3), self-monitoring of outcome(s) of behavior (2.4), and feedback on outcome(s) of behavior (2.7) [54]. Activity calendars consisted of tables with columns for 7 days, with each column sectioned to indicate morning, noon, and evening times. During the guided intervention sessions (face-to-face and phone-based), participants were asked to fill in the day and date (headers) and their current PA-specific action plans (i.e., cues and behavior). The final two activity calendars also asked participants to indicate with whom they planned to pursue an activity. Calendars were completed by participants daily at the end of each day throughout weeks 1 to 4, weeks 25 to 28, and weeks 50 to 53 following M0. For the activity-calendar periods, participants were asked to put a checkmark next to each action plan they had implemented as planned on a given day. At the bottom of the calendar-columns, participants could enter additional and/or alternative activities pursued during a given day. Completed sheets were sent back to the study center (see [8] for a more detailed description of these intervention components).

### Measures

The present article used data relevant for the analysis of the pre-registered primary research question under study [8], these include M0, one week following M0, M12, and M24.

#### OAK symptoms

The primary endpoint was self-reported OAK symptoms at M24 assessed with the WOMAC in its version for OAK administered in German [7]. The WOMAC is a validated and internationally used questionnaire which comprises 24 items with three subscales of OAK symptoms, i.e., OAK functional limitations (17 items), OAK pain (5 items), and OAK stiffness (2 items), to which participants responded on 11-point scales ranging from 0 to 10. Item missings were imputed with item means and responses were summed ranging from 0 to 240 (WOMAC total score), 0 to 170 (WOMAC-functional limitations), 0 to 50 (WOMAC-pain), and 0 to 20 (WOMAC-stiffness) [38]. Higher values indicated higher-levels of OAK symptoms. M0 and M24 indicators were used in the present analyses. Internal consistencies were medium to high, with Cronbach’s alphas α = 0.95 (M0) and α = 0.96 (M24) for the overall score of OAK symptoms, α = 0.94 (M0) and α = 0.95 (M24) for WOMAC-functional limitations, α = 0.79 (M0) and α = 0.88 (M24) for WOMAC-pain, and α = 0.80 (M0) and α = 0.86 (M24) for WOMAC-stiffness.

#### Moderate-to-vigorous physical activity (MVPA)

Daily MVPA (in minutes) averaged over one week as assessed with tri-axial accelerometer devices (ActiGraph GT3X, Pensacola, Fl) at M0 and M12 were used in the present analyses. Participants were instructed to wear the devices at their right hip during waking hours for one week at each assessment period. Using an algorithm by Sasaki et al. [55], minutes of MVPA were calculated for participants who had worn their accelerometers on at least 4 days for at least 10 h a day. Univariate outliers of MVPA (*z* >|3.29|) were substituted by values one unit higher/lower compared to the next most extreme value in the distribution [56].

#### HAPA-proposed volitional precursors of MVPA change

All HAPA-defined volitional precursors of MVPA change addressed in the PrevOP-PAP intervention were included in the manipulation checks, including assessments at M0 and M24. They were adapted from prior research [22, 23, 29, 41] and assessed specifically for the PA domain. Participants responded on 6-point scales ranging from 1 “does not apply at all/very unlikely” to 6 “applies exactly/highly likely”. Action planning was assessed with 4 items (M0 α = 0.97; M24 α = 0.98) and coping planning was measured with 5 items (M0 α = 0.94; M24 α = 0.94). Maintenance self-efficacy (M0 α = 0.84; M24 α = 0.79) and recovery self-efficacy (M0 α = 0.93; M24 α = 0.92) were measured with 3 items each. Action control was assessed with 6 items at M0 (α = 0.91) and M24 (α = 0.90). Finally, collaborative implementation intentions with a training partner (adapted from [29, 41]) were assessed with 4 items (M0 α = 0.98; M24 α = 0.99). For each HAPA-defined construct, we computed mean scores ranging from 1 to 6.

#### Behavioral intentions and covariates

Behavioral intentions as assessed at M0 and one week after the motivational treatment received by all participants were measured with 4 items (M0 α = 0.81; one week after M0 α = 0.76; [22, 23]). Covariates included baseline variables (M0) for which randomization failed or those that were associated with dropout. These included positive outcome expectancies (assessed with 6 items; α = 0.82; [22, 23]), negative affect as a source of self-efficacy (assessed with 2 items; α = 0.92; e.g., “Just before I start physical activities, I feel tired” [8, 57]), a visual analogue scale of pain (VAS-pain, 10 cm) in the knee on the M0 day (ranging from 0: “no pain” to 10: “strongest conceivable pain”) [58], and being divorced (one item), as assessed at M0. Additional covariates were body mass index (BMI; objectively assessed at M0), sex, and age which together with all other socio-demographic variables (Table 1) were assessed via self-report at M0. Unless 1-item assessments were used, items were averaged to a total mean score ranging from 1 “does not apply at all/very unlikely” to 6 “applies exactly/highly likely”.

### Statistical analyses

Analyses were conducted using R Statistical Software (v4.2.2 [59]). Randomization checks and drop-out analyses with all self-report and PA M0-assessments (see [8]) were done using analyses of variance for continuous variables and chi-square tests for nominal and ordinal-scale data. In case of several, potentially inter-related, randomization or drop-out mechanisms, these were followed up with logistic regression analyses predicting intervention group membership (coded 1; active control group membership coded 0) or drop-out status, respectively, to determine unique associations.

To benefit from full-information maximum likelihood procedures to retain all available data in models and perform analyses with an intent-to-treat approach [60], all other analyses were conducted as manifest models using the lavaan R package (v0.6–12 [61]). To examine intention change following the brief motivational treatment, a simple latent change score model, mimicking a paired samples *t*-test was conducted [62]. For manipulation checks of the HAPA-proposed volitional precursors of behavior change and to test simple effects of the PrevOP-PAP intervention group membership (coded 1; active control group membership coded 0) on OAK symptoms (WOMAC) at M24 as well as on MVPA at M12, manifest regression analyses were fit, regressing the respective M24 (or M12) indicator on the intervention condition as well as on its M0 counterpart. To test the primary hypothesis, a manifest path model was fit with intervention condition (PrevOP-PAP intervention group coded 1; active control group coded 0), M0 indicators of OAK symptoms (WOMAC) and MVPA, and M0 covariates as predictors, MVPA at M12 as a proposed mediator, and OAK symptoms (WOMAC) at M24 as the outcome. To test the predicted indirect effect, we used bias-corrected bootstrapping with 5,000 resamples [63].

Sensitivity analyses for the primary hypothesis test included several groups of covariates: BMI (range in this sample: 19.16 to 45.79 kg/m^2^), sex (0 = female, 1 = male) and age in years; randomization failures (VAS-pain in the knee today; divorced: 0/1); dropout mechanisms (positive outcome expectancies; negative affect as a source of self-efficacy); and dummy-coded medical PrevOP-MMT-conditions (PrevOP-MMT high-impact exercise condition, coded 1, PrevOP-MMT low-impact exercise condition, coded 1, with PrevOP-MMT active control condition being the reference group, coded 0). Continuous covariates were grand-mean centered.

As indicated in the study protocol [8], in preliminary analyses, we ascertained that dummy-coded PrevOP-MMT-conditions did not moderate the association between the proposed mediator (MVPA at M12) and primary outcome (OAK symptoms (WOMAC) at M24). No interactions emerged, hence PrevOP-MMT-conditions were included as simple-effect covariates in all sensitivity analyses [8]. As models including all covariates did not converge when using bias-corrected bootstrapping, the Sobel test was used to test predicted indirect effects in sensitivity analyses [64]. All manifest path models were fully saturated; thus, no model fit indices could be determined.

## Results

### Attrition analyses and randomization check

Participants dropping out before M24 (*n* = 69) and continuing participants (*n* = 172) were similar in most of the variables under study. However, participants dropping out before M24 reported higher levels of positive outcome expectancies, *t*(238) = 2.31, *p* = 0.022, *d* = 0.33, and higher levels of negative affect as a source of self-efficacy, *t*(99.73) = 2.29, *p* = 0.024, *d* = 0.35, at baseline. Randomization checks indicated no significant baseline differences between the PrevOP-PAP intervention condition and PrevOP-PAP active control condition, except for levels of pain in the knee (VAS-pain) on the M0 day being lower in the PrevOP-PAP intervention condition, *t*(234) = -2.30, *p* = 0.022, *d* = 0.30, and more participants being divorced in the PrevOP-PAP active control condition than in the PrevOP-PAP intervention condition, χ^2^ (1) = 7.00, *p* = 0.008, *V* = 0.17.

### Manipulation checks

Statistics of the central study variables and between-group differences are displayed in Table 2. For fully controlled multiple regression models testing simple effects of the PrevOP-PAP intervention condition (vs. PrevOP-PAP active control condition) see also additional file (Additional Table 1). Manipulation checks revealed significant increases in participants’ intentions to engage in regular MVPA at one week following the brief motivational intervention (*b* = 0.17, *SE* = 0.07, 95% CI [0.04; 0.30], *p* = 0.009). Controlling for M0, participants in the PrevOP-PAP intervention condition reported higher levels of action planning at M24 when compared to those in the PrevOP-PAP active control condition (*b* = 0.64, *SE* = 0.26, 95% CI [0.14; 1.15], *p* = 0.013). However, no further group differences in long-term increases in HAPA-proposed volitional precursors of MVPA change, including coping planning, maintenance self-efficacy and recovery self-efficacy, action control, and collaborative implementation intentions, emerged. As for collaborative implementation intentions as an indicator of network formation, which was optional, only few participants (27% of the PrevOP-PAP intervention condition) actually chose to consider being physically active together with a network member. Sensitivity analyses including all further covariates revealed the same pattern of results (additional file: Additional Table 1).

**Table 2:** Descriptive statistics of central variables

	**PrevOP-PAP intervention condition (*n* = 123)**	**PrevOP-PAP active control condition (*n* = 118)**	**Difference between PrevOP-PAP intervention and PrevOP-PAP active control condition**
Variable [scale’s range]	*M (SD)*	*M (SD)*	Est* (SE)* 95% CI
Maintenance self-efficacy [1-6]			
M0	4.28 (1.26)	4.55 (1.03)	
M24	4.19 (1.16)	3.98 (1.07)	0.24 (0.17) [-0.08; 0.57] ΔR^2^ = .01
Recovery self-efficacy [1-6]			
M0	4.88 (1.09)	5.13 (0.89)	
M24	4.75 (1.12)	4.54 (0.97)	0.25 (0.16) [-0.06; 0.56] ΔR^2^ = .01
Action planning [1-6]			
M0	3.96 (1.77)	3.99 (1.76)	
M24	4.27 (1.73)	3.65 (1.87)	0.64 (0.26)* [0.14; 1.15] ΔR^2^ = .03
Coping planning [1-6]			
M0	2.97 (1.58)	3.04 (1.59)	
M24	3.10 (1.52)	3.20 (1.47)	-0.10 (0.20) [-0.50; 0.31] ΔR^2^ = .00
Action control [1-6]			
M0	3.22 (1.45)	3.38 (1.39)	
M24	3.52 (1.34)	3.36 (1.33)	0.17 (0.18) [-0.20; 0.53] ΔR^2^ = .00
Collaborative implementation intentions [1-6]^a^			
M0	3.19 (2.12)	3.29 (2.08)	
M24	3.69 (2.10)	3.35 (2.12)	0.26 (0.44) [-0.60; 1.12] ΔR^2^ = .00
Moderate-to-vigorous physical activity (MVPA)			
M0	46.44 (29.11)	48.42 (28.21)	
M12	40.69 (25.83)	49.64 (29.25)	-3.45 (3.11) [-9.55; 2.65] ΔR^2^ = .00
OAK Symptoms (WOMAC) [0–240]			
M0	71.76 (33.35)	75.87 (39.28)	
M24	43.23 (32.21)	54.96 (37.43)	-9.30 (4.74)^†^ [-18.58; -0.02] ΔR^2^ = .02
WOMAC-functional limitations [0–170]			
M0	47.98 (24.79)	50.77 (29.66)	
M24	29.10 (22.12)	37.01 (26.76)	-6.32 (3.30)^†^ [-12.79; 0.15] ΔR^2^ = .01
WOMAC-pain [0–50]			
M0	15.79 (7.91)	16.52 (8.38)	
M24	8.94 (7.87)	12.30 (9.49)	-2.77 (1.17)* [-5.05; -0.49] ΔR^2^ = .02
WOMAC-stiffness [0–20]			
M0	7.98 (4.50)	8.58 (4.53)	
M24	5.19 (4.03)	5.66 (3.89)	-0.28 (0.56) [-1.39; 0.82] ΔR^2^ = .00

147 ≤ *n* ≤ 241 due to missing values unless otherwise noted

*M* Mean, *SD* Standard deviation, *Est.* Estimate, *SE* Standard error, *CI* Confidence interval, *OAK* Osteoarthritis of the knee, *M0* Baseline, *M12* 12-months follow-up, *M24* 24-months follow-up, *PrevOP-PAP* PrevOP-Psychological Adherence Program, *WOMAC* Western Ontario and McMaster Universities Osteoarthritis Index [7]

^a^Based on *n* = 128 (M0) and *n* = 88 (M24) as many participants decided not to participate in network formation intervention and thus did not provide data. Between-condition differences as indicated by manifest regression analyses regressing the respective M24 (or M12) indicator on its M0 counterpart and intervention condition (PrevOP-PAP intervention condition, coded 1, vs. PrevOP-PAP active control condition, coded 0); coefficients are unstandardized. ΔR^2^ = Incremental variance explained in the respective M24 (or M12) outcome, controlled for its M0 counterpart, when entering the intervention condition (PrevOP-PAP intervention condition, coded 1, vs. PrevOP-PAP active control condition, coded 0) in the manifest regression model

^†^ *p* < .10; * *p* < .05

### Indirect effects of the PrevOP-PAP intervention on OAK symptoms via MVPA

Results of manifest path models predicting participants’ OAK symptoms (WOMAC) at 24 months with MVPA at 12 months as a mediator are displayed in Table 3. Controlling for M0 OAK symptoms (WOMAC) and MVPA, no indirect effect of the PrevOP-PAP intervention on participants’ OAK symptoms (WOMAC, at 24 months) via MVPA at 12 months emerged, when compared to the PrevOP-PAP active control condition. At the end of the active intervention phase (12 months post study entry) and controlling for M0, participants in the PrevOP-PAP intervention condition did not differ from those in the PrevOP-PAP active control condition with regard to MVPA (Table 3; for multiple regression models testing simple effects see additional file (Additional Table 1)). At 24 months, participants in the PrevOP-PAP intervention condition reported decreased levels of OAK symptoms (WOMAC) when compared to participants in the PrevOP-PAP active control condition (*b* = -9.81, *SE* = 4.77, 95% CI [-19.51; -0.66], *p* = 0.040). However, in sensitivity analyses this effect did not remain statistically significant (*p* = 0.071; see Model 2, Table 3; for multiple regression models testing simple effects, see additional file, Additional Table 1).

**Table 3:** Manifest path models predicting participants‘ symptoms of Osteoarthritis of the Knee (OAK symptoms)

	**Model 1** **Dependent variable: OAK symptoms (WOMAC) at M24**			**Model 2** **Dependent variable: OAK symptoms (WOMAC) at M24**		
	**Direct Effect**		**Indirect Effect**	**Direct Effect**		**Indirect Effect**
	MVPA at M12	OAK symptoms (WOMAC) at M24	EV → M → DV	MVPA at M12	OAK symptoms (WOMAC) at M24	EV → M → DV
	Est (*SE*) 95% CI	Est (*SE*) 95% CI	Est (*SE*) 95% CI	Est (*SE*) 95% CI	Est (*SE*) 95% CI	Est (*SE*) 95% CI
PrevOP-PAP intervention condition (vs. PrevOP-PAP active control condition)	-3.78 (3.42) [-10.80; 2.58]	-9.81 (4.83)* [-19.18; -0.40]	0.43 (0.89) [-0.44; 3.80]	-3.29 (3.05) [-9.27; 2.69]	-8.41 (4.66)^†^ [-17.53; 0.71]	0.32 (0.55) [-0.77; 1.40]
**Mediator:**						
MVPA at M12		-0.11 (0.15) [-0.42; 0.19]			-0.10 (0.14) [-0.38; 0.19]	
**Covariates:**						
OAK symptoms (WOMAC) at M0	-0.06 (0.05) [-0.16; 0.02]	0.46 (0.09)*** [0.29; 0.64]	0.01 (0.01) [-0.01; 0.05]	-0.05 (0.06) [-0.16; 0.07]	0.31 (0.09)*** [0.14; 0.48]	0.00 (0.01) [-0.01; 0.02]
MVPA at M0	0.72 (0.08)*** [0.57; 0.86]	0.07 (0.17) [-0.25; 0.40]	-0.08 (0.11) [-0.31; 0.13]	0.71 (0.06)*** [0.60; 0.82]	0.03 (0.14) [-0.23; 0.30]	-0.07 (0.10) [-0.27; 0.13]
PrevOP-MMT high-impact exercise condition (vs. PrevOP-MMT active control condition)				0.01 (3.74) [-7.32; 7.35]	0.31 (5.66) [-10.78; 11.40]	-0.00 (0.36) [-0.71; 0.70]
PrevOP-MMT low-impact exercise condition (vs. PrevOP-MMT active control condition)				0.37 (3.71) [-6.90; 7.64]	-1.39 (5.74) [-12.65; 9.86]	-0.04 (0.36) [-0.75; 0.67]
Sex (male vs. female)				0.86 (3.13) [-5.27; 6.99]	0.04 (4.79) [-9.35; 9.43]	-0.08 (0.33) [-0.73; 0.56]
Age				-0.25 (0.21) [-0.67; 0.16]	-0.16 (0.34) [-0.83; 0.51]	0.02 (0.04) [-0.06; 0.11]
Body mass index				-0.48 (0.35) [-1.16; 0.20]	1.30 (0.51)* [0.29; 2.31]	0.05 (0.08) [-0.11; 0.20]
Divorced (vs. not divorced)				8.01 (3.96)* [0.24; 15.78]	-5.28 (6.35) [-17.72; 7.15]	-0.77 (1.23) [-3.18; 1.64]
VAS-pain				0.21 (1.04) [-1.83; 2.25]	3.41 (1.57)* [0.34; 6.48]	-0.02 (0.11) [-0.23; 0.19]
Positive outcome expectancies				-2.06 (1.75) [-5.49; 1.37]	-0.66 (2.70) [-5.95; 4.63]	0.20 (0.35) [-0.48; 0.88]
Source of self-efficacy: negative affect				0.55 (1.50) [-2.38; 3.49]	-1.34 (2.31) [-5.86; 3.18]	-0.05 (0.17) [-0.38; 0.27]
***R***^**2**^	MVPA at M12: *R*^2^ = 0.57; OAK symptoms at M24: *R*^2^ = 0.26			MVPA at M12: *R*^2^ = 0.60; OAK symptoms at M24: *R*^2^ = 0.32		

*N* = 241 participants. Because manifest path-models were fully saturated, no model fit could be determined. Unstandardized coefficients. Dichotomous covariates (coded 1/0): PrevOP-PAP intervention condition (coded 1, vs. PrevOP-PAP active control condition, coded 0), PrevOP-MMT high- impact exercise condition (coded 1, vs. PrevOP-MMT active control condition, coded 0), PrevOP-MMT low-impact exercise condition (coded 1, vs. PrevOP-MMT active control condition, coded 0), sex male (coded 1, vs. female, coded 0), divorced (coded 1, vs. not divorced, coded 0). Continuous covariates (grand-mean centered, per one point increase): OAK symptoms (WOMAC) at M0, MVPA at M0, age in years, body mass index, VAS-pain, positive outcome expectancies, source of self-efficacy: negative affect

*Est.* Estimate, *SE* Standard error, *CI* Confidence interval, *EV* Exogenous variable, *M* Mediator, *DV* Dependent variable, *MVPA* Moderate to Vigorous Physical Activity, *M0* Baseline, *M12* 12-months follow-up, *M24* 24-months follow-up, *PrevOP-PAP* PrevOP-Psychological Adherence Program, *PrevOP-MMT* PrevOP-Main Medical Trial, *VAS* Visual analogue scale, *WOMAC* Western Ontario and McMaster Universities Osteoarthritis Index [7]

^†^ *p* < .10; * *p* < .05; ** *p* < .01; *** *p* < .001

Exploratory follow-up analyses with different domains of OAK symptoms (WOMAC-pain, WOMAC-functional limitations, WOMAC-stiffness) as outcomes and controlling for M0 levels revealed that participants in the PrevOP-PAP intervention condition reported lower levels of WOMAC-pain at 24 months post study entry when compared to the PrevOP-PAP active control condition (additional file: Additional Table 1). Sensitivity analyses revealed the same pattern of results. With regard to WOMAC-functional limitations and WOMAC-stiffness, no intervention effects emerged. Again, and consistent with results regarding overall OAK symptoms (WOMAC), no indirect effects of PrevOP-PAP via MVPA at 12 months on WOMAC-pain, -functional limitations, or -stiffness were found (additional file: Additional Tables 2, 3, and 4). All manifest path models were fully saturated.

## Discussion

This primary analysis report evaluated outcomes of the psychological adherence program PrevOP-PAP that was designed to enhance PA and reduce OAK symptoms (WOMAC) among patients with moderate OAK. The intervention program PrevOP-PAP adopted motivational, volitional, and networking intervention strategies based on the HAPA to support OAK patients’ uptake and maintenance of regular MVPA and reduce OAK symptoms (WOMAC). Intervention effects were contrasted with the PrevOP-PAP active control condition, in which participants only received the motivational intervention. As the primary hypothesis, it was assumed that participants of the PrevOP-PAP intervention condition (compared with participants of the PrevOP-PAP active control condition) would engage in more MVPA at the end of the active intervention phase which would then translate to lower levels of OAK symptoms (WOMAC) at the end of the study period. Present findings did not confirm the proposed intervention effects on overall OAK symptoms (WOMAC) or MVPA. Moreover, MVPA did not mediate the association between the intervention and OAK symptoms (WOMAC).

### Indirect effects of the PrevOP-PAP intervention on OAK symptoms via MVPA

Compared to the control group, intervention effects on overall OAK symptoms (WOMAC) trended towards a decrease at the end of the study period. Contrary to our hypothesis, this effect did no longer reach statistical significance in sensitivity analyses. Still, exploratory follow-up analyses with different domains of OAK symptoms (WOMAC) as outcomes (i.e., WOMAC-pain, WOMAC-functional limitations, and WOMAC-stiffness) revealed effects of the PrevOP-PAP intervention on pain, but null effects on functional limitations and stiffness at 24 months following study entry. These findings resemble meta-analytic evidence on self-management education programs for osteoarthritis suggesting small – mostly shorter-term – improvements in pain, but no beneficial effects on physical functioning when compared to control groups [38, 65]. However, underlying intervention processes with regard to the effect on WOMAC-pain remain unclear. As our findings indicated null effects of the PrevOP-PAP intervention on levels of MVPA at the end of the intervention period, MVPA did not serve as the proposed mediator. Similar findings with positive effects of a motivational interviewing-based intervention on OAK symptoms, but null effects on MVPA have been reported elsewhere [66]. Possibly, participants of the PrevOP-PAP intervention condition learned over time how to better accept their levels of pain, which has been shown to be associated with lower levels of pain intensities [67].

### Effects of the PrevOP-PAP intervention on MVPA and its HAPA-proposed volitional precursors

Importantly, the question arises why participants did not increase their MVPA during the intervention period. On the one hand, increases in intentions to engage in regular MVPA one week after the motivational intervention indicated a successful motivational manipulation for all participants. However, manipulation checks of the PrevOP-PAP intervention yielded only one effect on the HAPA-proposed volitional precursors of MVPA change at the end of the study period. At 24 months following study entry, only action planning showed a significant increase in the PrevOP-PAP intervention condition (vs. PrevOP-PAP active control condition). The finding on intervention effects for action planning is in line with prior findings in the context of cardiac and orthopedic rehabilitation [45] and osteoarthritis albeit for shorter time frames [38]. However, prior findings also suggest that action planning as a stand-alone volitional strategy may not be sufficient to facilitate the uptake and maintenance of MVPA in the long-term [68].

In this context, coping planning and action control have been highlighted as important additional key intervention components to enhance the effects of action planning [39]. However, in PrevOP-PAP, manipulation checks revealed no significant between-group differences in coping planning and action control at 24 months following study entry. As a possible explanation for the null effect regarding coping planning, action plans formed during the PrevOP-PAP intervention might already have been of high enough quality, as patients also planned increases in physical activities, they were already familiar with. This may have resulted in less need for additional coping plans, and led to maintenance rather than increases in levels of coping planning. Moreover, participants of the PrevOP-PAP intervention condition reported decreased levels of pain as an OAK-specific barrier to physical activity over time. This reduction in barriers to become more active may have also contributed to a maintenance in levels of coping planning, rather than their expected increase.

Regarding the null effect on action control, three one-month paper–pencil activity calendar phases delivered throughout the PrevOP-PAP intervention with extended periods of no intervention delivery might not have been sufficient to foster action control in the long run. Future research could implement m-health self-monitoring applications which are permanently available to facilitate continuous and long-term action control.

Furthermore, the overall moderate OAK severity and prolonged disease duration in our sample as well as a decrease in pain in the intervention condition might further explain null effects of the PrevOP-PAP intervention on changes in self-efficacy. With decreasing salience of barriers or decreasing barriers, such as pain, over time, increases in behavior-specific self-efficacy become less likely, because self-efficacy is always measured up against perceived difficulties or barriers to act. Also, with regard to the social network formation indicator, i.e., collaborative implementation intentions, the PrevOP-PAP intervention did not yield significant intervention effects and many participants of the intervention condition decided not to participate in the optional network creation intervention. As vicarious experiences and positive affective states experienced in social networks serve as sources of self-efficacy, this might also have affected levels of self-efficacy in our sample [10, 69]. Again, the development of m-health applications with features to digitally create social networks may be beneficial to foster network formation in the long run. Moreover, future interventions could focus on perceived enjoyment with PA, done alone or with others, which has been shown to be a strong correlate of PA among patients with osteoarthritis [10] and might also strengthen the network formation intervention [70, 71].

On the other hand, when interpreting null effects on MVPA, it must be noted that participants demonstrated relatively high baseline levels of MVPA (i.e., on average around 47 min per day) when compared to previously reported levels of MVPA among patients with OAK [72]. Thus, participants’ capacity for additional increases in MVPA throughout the study period might have been limited. Moreover, intervention programs using self-regulatory strategies such as action planning were shown to be less effective among individuals who were already physically active when compared to sedentary populations [73]. At the same time, higher levels of MVPA at baseline might also reflect some reactivity to the measurement via accelerometery [74]. Future studies should consider extended periods of baseline measurement with accelerometry in order to prevent measurement reactivity.

### Strengths and limitations

This study has several strengths. First, this RCT included a long follow-up period up to 24 months post study entry (i.e., 12 months following the intervention period) to elucidate causal mechanisms of the PrevOP-PAP intervention in the long term. The intervention program PrevOP-PAP was based on theoretically-derived health behavior change techniques to allow for testing underlying processes of behavior change [14, 54]. The computer-assisted intervention facilitated a standardized delivery of the PrevOP-PAP intervention program. Moreover, in this RCT, MVPA was objectively assessed using accelerometers. This may have reduced problems often associated with MVPA self-reports such as recall biases or mere measurement effects due to repeated active assessments [75, 76].

However, some limitations must be acknowledged. Despite the advantages of mediation analyses to understand the causal mechanisms of this complex intervention over time, this modelling approach also comes with drawbacks. Given the complexity of OAK symptoms, it seems likely that further non-hypothesized factors may have explained changes in OAK symptoms over time which, however, were not captured using this theory-guided approach. Future research may apply data-driven approaches such as Bayesian Networks to further elucidate intervention mechanisms of the PrevOP-PAP intervention [77].

Second, intervention delivery and data analyses in this RCT were unmasked due to ethical and practical reasons. Participants were informed that they would be randomly allocated to either the PrevOP-PAP intervention condition or the PrevOP-PAP active control condition and study personnel were aware of delivering the intervention treatment. Masking of statistical analysis is desirable for future RCT evaluation.

Third, attrition rates were elevated across two years of the study period (29%) with the majority of participants dropping out during the intervention period within the first year. This may be explained by a high participant burden due to intensive intervention delivery and repeated measurements in crossed psychological PrevOP-PAP and medical PrevOP-MMT trials. However, it must be noted that the sample size of completers in PrevOP-PAP was sufficient to detect the proposed mediation effect with a power of 0.80 as the overall sample size estimation was higher for the PrevOP-MMT. Moreover, we aimed to reduce potential selection effects due to attrition by conducting intent-to-treat analyses with full maximum likelihood estimation and considering potential dropout mechanisms as covariates in our sensitivity analyses [60].

Fourth, despite numerous advantages of the objective assessment of PA, this approach may also have drawbacks. Whereas participants may have increased their engagement in joint-friendly MVPA that are particularly recommended in the context of OAK such as swimming or riding a bike [6], hip-worn accelerometers cannot capture these specific types of PA. Thus, participants’ levels of MVPA may have been underestimated. Lastly, the active control group and the crossed study design with structured exercise conditions may have caused overall higher levels of MVPA throughout the study period both in the PrevOP-PAP intervention condition and the PrevOP-PAP active control condition. This might have further limited the variance in levels of MVPA.

### Conclusions

This psychological adherence program was based on HAPA-derived behavior change techniques and specifically designed for patients with moderate OAK to facilitate the uptake and maintenance of physical activity. Whereas levels of action planning significantly increased following the intervention, primary analyses did not yield beneficial effects of the PrevOP-PAP intervention on physical activity and limited effects on OAK symptoms (WOMAC), i.e., only a decrease in WOMAC-pain at the end of the study period emerged in exploratory follow-up analyses. Resembling meta-analytic findings on self-management programs for OAK, the PrevOP-PAP intervention might thus appear promising for improved disease management (e.g., coping with pain). However, as physical activity did not serve as a mediator of this exploratory finding, underlying mechanisms of improvements in pain still remain unclear. Future research should further investigate which intervention components of the PrevOP-PAP specifically targeted the patients’ pain management. Subsequently, the intervention program could be refined and provided as an m-health application on a large scale for patients with OAK.

## Supplementary Information

**Additional file 1: Additional Information 1.** Inclusion and Exclusion Criteria. **Additional Figure 1.** Conditions of the PrevOP-Main Medical Trial (PrevOP-MMT) nested in the PrevOP-Psychological Adherence Program (PrevOP-PAP) conditions. **Additional Table 1.** Manifest Regression Analyses Predicting Central Variables. **Additional Table 2.** Manifest Path Models Predicting Participants‘ Functional Limitations Associated with Osteoarthritis of the Knee. **Additional Table 3.** Manifest Path Models Predicting Participants‘ Pain Associated with Osteoarthritis of the Knee. **Additional Table 4.** Manifest Path Models Predicting Participants‘ Stiffness Associated with Osteoarthritis of the Knee.

## Data Availability

The datasets generated and analyzed during the current study are available from the corresponding author upon request.

## Abbreviations

BCT: Behavior Change Technique
BMI: Body Mass Index
CONSORT: Consolidated Standards of Reporting Trials
HAPA: Health Action Process Approach
M: “Month”
MVPA: Moderate-to-Vigorous Physical Activity
PA: Physical Activity
PAP: Psychological Adherence Program
PrevOP: PREVenting the impairment of primary Osteoarthritis by high-impact long-term Physical exercise regimen
PrevOP-MMT: PrevOP-Main Medical Trial
PrevOP-PAP: PrevOP-Psychological Adherence Program
OAK: OsteoArthritis of the Knee
RCT: Randomized Controlled Trial
TIDieR: Template for Intervention Description and Replication
WOMAC: Western Ontario and McMaster Universities Osteoarthritis Index
