# Supplementary material for: Enhancing physical activity and reducing symptoms of patients with osteoarthritis of the knee: a randomized controlled trial of the PrevOP-Psychological Adherence Program
Source: BMC Musculoskelet Disord. 2023 Jul 4;24:550. doi: 10.1186/s12891-023-06661-x (PMC10318642; doi:10.1186/s12891-023-06661-x)
Supplement: Supplementary file 1 — Additional file 1: Additional Information 1. Inclusion and Exclusion Criteria. Additional Figure 1. Conditions of the PrevOP-Main Medical Trial (PrevOP-MMT) nested in the PrevOP-Psychological Adherence Program (PrevOP-PAP) conditions. Additional Table 1. Manifest Regression Analyses Predicting Central Variables. Additional Table 2. Manifest Path Models Predicting Participants‘ Functional Limitations Associated with Osteoarthritis of the Knee. Additional Table 3. Manifest Path Models Predicting Participants‘ Pain Associated with Osteoarthritis of the Knee. Additional Table 4. Manifest Path Models Predicting Participants‘ Stiffness Associated with Osteoarthritis of the Knee. [file 12891_2023_6661_MOESM1_ESM.docx]

**Additional file**

**Enhancing Physical Activity and Reducing Symptoms of Patients with Osteoarthritis of the Knee: A Randomized Controlled Trial of the PrevOP-Psychological Adherence Program**

**Content**

|  | **Content** | **Page** |
| --- | --- | --- |
| Additional Information 1 | Inclusion and Exclusion Criteria | 3 |
| Additional Figure 1 | Conditions of the PrevOP-Main Medical Trial (PrevOP-MMT) nested in the PrevOP-Psychological Adherence Program (PrevOP-PAP) conditions | 4 |
| Additional Table 1 | Manifest Regression Analyses Predicting Central Variables | 5 |
| Additional Table 2 | Manifest Path Models Predicting Participants‘ Functional Limitations Associated with Osteoarthritis of the Knee (OAK functional limitations) | 13 |
| Additional Table 3 | Manifest Path Models Predicting Participants‘ Pain Associated with Osteoarthritis of the Knee (OAK pain) | 15 |
| Additional Table 4 | Manifest Path Models Predicting Participants‘ Stiffness Associated with Osteoarthritis of the Knee (OAK stiffness) | 17 |
| References | References | 19 |

**Additional Information 1: Inclusion and Exclusion Criteria**

Inclusion criteria: ambulatory persons with OAK (radiographical signs of grades 2 and 3 following the Kellgren and Lawrence classification [1]); between 40 and 80 years; with pain on at least 50% of days of the past month (>40 mm [2]); with joint space width of 2.5 to 5 mm with predominant knee osteoarthritis of the medial tibiofemoral compartment; and written informed consent [3].

Exclusion criteria: Knee (any time) or hip (past 12 months) total endoprothesis; participation in other clinical trials, cognitive impairment, insufficient German language skills; any of the following comorbidities during the past 5 years: cancer, angina pectoris, coronary intervention, thromboembolic events (acute deep venous thrombosis, pulmonary embolism, stroke, myocardial infarction, arrhythmia absoluta, uncontrolled hypertension, retinal artery occlusion, vena centralise retinae occlusion); any of the following comorbidities and states: paraplegia, hemiplegia, severe rheumatoid arthritis, severe polyneuropathy, severe vertebral disc prolapse, severe spinal canal stenosis, dementia, acute tendinitis (legs only), current fractures (pelvic and legs), cholecystolithiasis, kidney stones, urethral stones, hernia abdominalis and/or inguinalis, acute migraine, current wounds (pelvis and legs), epilepsy, pregnancy; also recent intra-articular injection (notably glucocorticoids <3 months previously or hyaluronic acid <6 months previously), clinical deformities, and previous treatments acting on cartilage (oral glucosamine ≥1500 mg/day and chondroitin sulphate <3 months previously).

**Additional Figure 1**

*Conditions of the PrevOP-Maint Medical Trial (PrevOP-MMT) nested in the PrevOP-Psychological Adherence Program (PrevOP-PAP) conditions*

PrevOP-MMT high-exercise +

PrevOP-PAP intervention

*n* = 43

PrevOP-MMT high-exercise +

PrevOP-PAP active control

*n* = 38

PrevOP-MMT low-exercise +

PrevOP-PAP intervention

*n* = 41

PrevOP-MMT low-exercise +

PrevOP-PAP active control

*n* = 41

PrevOP-MMT active control +

PrevOP-PAP intervention

*n* = 39

PrevOP-MMT active control +

PrevOP-PAP active control

*n* = 39

PrevOP-PAP intervention

*n* = 123

PrevOP-PAP active control

*n* = 118

**Additional Table 1**

*Manifest Regression Analyses Predicting Central Variables*

|  | **Model 1** | **Model 2** |
| --- | --- | --- |
|  | Est *(SE)*  95% CI | Est *(SE)*  95% CI |
| **DV: Maintenance self-efficacy at M24** |  |  |
| PrevOP-PAP intervention condition  (vs. PrevOP-PAP active control condition) | 0.24 (0.17) [-0.08; 0.57] | 0.25 (0.17) [-0.07; 0.57] |
| Maintenance self-efficacy at M0 | 0.16 (0.07)* [0.02; 0.30] | 0.13 (0.07)^†^ [-0.01; 0.27] |
| PrevOP-MMT high-impact exercise condition (vs. PrevOP-MMT active control condition) |  | -0.42 (0.20)* [-0.82; -0.03] |
| PrevOP-MMT low-impact exercise condition (vs. PrevOP-MMT active control condition) |  | -0.50 (0.20)* [-0.90; -0.10] |
| Sex |  | 0.09 (0.17) [-0.25; 0.42] |
| Age |  | -0.00 (0.01) [-0.03; 0.02] |
| Body mass index |  | -0.03 (0.02)^†^ [-0.07; 0.00] |
| Divorced |  | -0.06 (0.22) [-0.49; 0.37] |
| VAS-pain |  | -0.02 (0.05) [-0.11; 0.06] |
| Positive outcome expectancies |  | 0.19 (0.10)* [0.00; 0.38] |
| Source of self-efficacy: negative affect |  | -0.01 (0.08) [-0.17; 0.15] |
| **DV: Recovery self-efficacy at M24** |  |  |
| PrevOP-PAP intervention condition  (vs. PrevOP-PAP active control condition) | 0.25 (0.16) [-0.06; 0.56] | 0.25 (0.15) [-0.05; 0.54] |
| Recovery self-efficacy at M0 | 0.21 (0.08)** [0.06; 0.36] | 0.06 (0.08) [-0.10; 0.22] |
| PrevOP-MMT high-impact exercise condition (vs. PrevOP-MMT active control condition) |  | 0.03 (0.19) [-0.33; 0.39] |
| PrevOP-MMT low-impact exercise condition (vs. PrevOP-MMT active control condition) |  | -0.15 (0.19) [-0.52; 0.22] |
| Sex |  | -0.04 (0.16) [-0.35; 0.26] |
| Age |  | -0.01 (0.01) [-0.04; 0.01] |
| Body mass index |  | -0.04 (0.02)* [-0.07; -0.01] |
| Divorced |  | 0.25 (0.20) [-0.14; 0.65] |
| VAS-pain |  | 0.00 (0.04) [-0.08; 0.09] |
| Positive outcome expectancies |  | 0.24 (0.09)** [0.07; 0.42] |
| Source of self-efficacy: negative affect |  | -0.09 (0.07) [-0.24; 0.05] |
| **DV: Action planning at M24** |  |  |
| PrevOP-PAP intervention condition  (vs. PrevOP-PAP active control condition) | 0.64 (0.26)* [0.14; 1.15] | 0.66 (0.25)** [0.17; 1.15] |
| Action planning at M0 | 0.34 (0.07)*** [0.20; 0.48] | 0.27 (0.07)*** [0.14; 0.41] |
| PrevOP-MMT high-impact exercise condition (vs. PrevOP-MMT active control condition) |  | 0.22 (0.30) [-0.37; 0.82] |
| PrevOP-MMT low-impact exercise condition (vs. PrevOP-MMT active control condition) |  | 0.35 (0.31) [-0.25; 0.96] |
| Sex |  | -0.20 (0.26) [-0.70; 0.31] |
| Age |  | 0.06 (0.02)*** [0.03; 0.10] |
| Body mass index |  | -0.04 (0.03) [-0.09; 0.01] |
| Divorced |  | 0.46 (0.33) [-0.19; 1.11] |
| VAS-pain |  | 0.06 (0.07) [-0.07; 0.20] |
| Positive outcome expectancies |  | 0.23 (0.15) [-0.06; 0.51] |
| Source of self-efficacy: negative affect |  | -0.12 (0.12) [-0.36; 0.12] |
| **DV: Coping planning at M24** |  |  |
| PrevOP-PAP intervention condition  (vs. PrevOP-PAP active control condition) | -0.10 (0.20) [-0.50; 0.31] | -0.09 (0.20) [-0.47; 0.30] |
| Coping Planning at M0 | 0.43 (0.07)*** [0.30; 0.56] | 0.35 (0.07)*** [0.22; 0.48] |
| PrevOP-MMT high-impact exercise condition (vs. PrevOP-MMT active control condition) |  | 0.73 (0.24)** [0.26; 1.20] |
| PrevOP-MMT low-impact exercise condition (vs. PrevOP-MMT active control condition) |  | 0.77 (0.24)** [0.29; 1.24] |
| Sex |  | -0.25 (0.20) [-0.64; 0.15] |
| Age |  | 0.04 (0.01)* [0.01; 0.07] |
| Body mass index |  | -0.00 (0.02) [-0.04; 0.04] |
| Divorced |  | -0.10 (0.26) [-0.61; 0.41] |
| VAS-pain |  | 0.11 (0.05)* [0.01; 0.22] |
| Positive outcome expectancies |  | 0.19 (0.11)^†^ [-0.03; 0.42] |
| Source of self-efficacy: negative affect |  | -0.08 (0.10) [-0.27; 0.11] |
| **DV: Action control at M24** |  |  |
| PrevOP-PAP intervention condition  (vs. PrevOP-PAP active control condition) | 0.17 (0.18) [-0.20; 0.53] | 0.18 (0.18) [-0.17; 0.54] |
| Action Control at M0 | 0.40 (0.06)*** [0.27; 0.52] | 0.33 (0.07)*** [0.20; 0.46] |
| PrevOP-MMT high-impact exercise condition (vs. active control condition) |  | 0.17 (0.22) [-0.27; 0.60] |
| PrevOP-MMT low-impact exercise condition (vs. active control condition) |  | 0.37 (0.22) [-0.07; 0.80] |
| Sex |  | -0.21 (0.19) [-0.58; 0.15] |
| Age |  | 0.04 (0.01)** [0.02; 0.07] |
| Body mass index |  | 0.00 (0.02) [-0.04; 0.04] |
| Divorced |  | 0.16 (0.24) [-0.31; 0.63] |
| VAS-pain |  | 0.05 (0.05) [-0.04; 0.15] |
| Positive outcome expectancies |  | 0.15 (0.11) [-0.05; 0.36] |
| Source of self-efficacy: negative affect |  | -0.12 (0.09) [-0.29; 0.05] |
| **DV: Collaborative implementation intentions at M24** |  |  |
| PrevOP-PAP intervention condition  (vs. PrevOP-PAP active control condition) | 0.26 (0.44) [-0.60; 1.12] | 0.02 (0.44) [-0.84; 0.89] |
| Collaborative implementations intention at M0 | 0.23 (0.13)^†^ [-0.02; 0.48] | 0.27 (0.13)* [0.02; 0.52] |
| PrevOP-MMT high-impact exercise condition (vs. PrevOP-MMT active control condition) |  | 0.40 (0.51) [-0.60; 1.39] |
| PrevOP-MMT low-impact exercise condition (vs. PrevOP-MMT active control condition) |  | 0.09 (0.56) [-1.00; 1.18] |
| Sex |  | -0.77 (0.46)^†^ [-1.68; 0.13] |
| Age |  | -0.02 (0.03) [-0.08; 0.04] |
| Body mass index |  | -0.00 (0.05) [-0.10; 0.09] |
| Divorced |  | -1.19 (0.60)* [-2.37; -0.00] |
| VAS-pain |  | 0.00 (0.12) [-0.23; 0.24] |
| Positive outcome expectancies |  | 0.09 (0.25) [-0.40; 0.58] |
| Source of self-efficacy: negative affect |  | -0.20 (0.23) [-0.65; 0.25] |
| **DV: Moderate-to-vigorous physical activity at M12** |  |  |
| PrevOP-PAP intervention condition (vs. PrevOP-PAP active control condition) | -3.45 (3.11) [-9.55; 2.65] | -3.34 (3.05) [-9.33; 2.64] |
| Moderate-to-vigorous physical activity at M0 | 0.72 (0.05)*** [0.61; 0.83] | 0.71 (0.06)*** [0.60; 0.82] |
| PrevOP-MMT high-impact exercise condition (vs. PrevOP-MMT active control condition) |  | 0.07 (3.74) [-7.27; 7.41] |
| PrevOP-MMT low-impact exercise condition (vs. PrevOP-MMT active control condition) |  | 0.21 (3.71) [-7.07; 7.49] |
| Sex |  | 0.72 (3.13) [-5.42; 6.85] |
| Age |  | -0.27 (0.21) [-0.68; 0.15] |
| Body mass index |  | -0.54 (0.33) [-1.20; 0.11] |
| Divorced |  | 8.02 (3.97)* [0.24; 15.79] |
| VAS-pain |  | -0.38 (0.84) [-2.03; 1.27] |
| Positive outcome expectancies |  | -2.01 (1.76) [-5.45; 1.43] |
| Source of self-efficacy: negative affect |  | 0.42 (1.49) [-2.51; 3.35] |
| **DV: OAK Symptoms (WOMAC) at M24** |  |  |
| PrevOP-PAP intervention condition  (vs. PrevOP-PAP active control condition) | -9.30 (4.74)* [-18.58; -0.02] | -7.98 (4.63)^†^ [-17.06; 1.09] |
| OAK Symptoms (WOMAC) at M0 | 0.47 (0.07)*** [0.34; 0.61] | 0.32 (0.09)*** [0.15; 0.49] |
| PrevOP-MMT high-impact exercise condition (vs. PrevOP-MMT active control condition) |  | 0.36 (5.66) [-10.74; 11.45] |
| PrevOP-MMT low-impact exercise condition (vs. PrevOP-MMT active control condition) |  | -1.23 (5.73) [-12.46; 10.00] |
| Sex |  | -0.14 (4.79) [-9.53; 9.25] |
| Age |  | -0.10 (0.33) [-0.75; 0.54] |
| Body mass index |  | 1.39 (0.50)** [0.41; 2.37] |
| Divorced |  | -5.98 (6.25) [-18.23; 6.26] |
| VAS-pain |  | 3.34 (1.55)* [0.30; 6.37] |
| Positive outcome expectancies |  | -0.56 (2.65) [-5.76; 4.64] |
| Source of self-efficacy: negative affect |  | -1.54 (2.27) [-5.99; 2.92] |
| **DV: WOMAC-functional limitations at M24** |  |  |
| PrevOP-PAP intervention condition  (vs. PrevOP-PAP active control condition) | -6.32 (3.30)^†^ [-12.79; 0.15] | -5.64 (3.23)^†^ [-11.98; 0.70] |
| WOMAC-functional limitations at M0 | 0.45 (0.06)*** [0.33; 0.58] | 0.35 (0.08)*** [0.20; 0.50] |
| PrevOP-MMT high-impact exercise condition (vs. PrevOP-MMT active control condition) |  | 0.13 (3.95) [-7.62; 7.88] |
| PrevOP-MMT low-impact exercise condition (vs. PrevOP-MMT active control condition) |  | 0.05 (4.00) [-7.79; 7.90] |
| Sex |  | 0.53 (3.34) [-6.03; 7.08] |
| Age |  | 0.04 (0.23) [-0.41; 0.50] |
| Body mass index |  | 1.02 (0.35)** [0.33; 1.70] |
| Divorced |  | -5.57 (4.36) [-14.12; 2.98] |
| VAS-pain |  | 1.80 (1.02)^†^ [-0.21; 3.80] |
| Positive outcome expectancies |  | -0.14 (1.86) [-3.79; 3.50] |
| Source of self-efficacy: negative affect |  | -1.43 (1.60) [-4.56; 1.70] |
| **DV: WOMAC-pain at M24** |  |  |
| PrevOP-PAP intervention condition  (vs. PrevOP-PAP active control condition) | -2.77 (1.17)* [-5.05; -0.49] | -2.25 (1.15)* [-4.49; -0.00] |
| WOMAC-pain at M0 | 0.53 (0.07)*** [0.39; 0.68] | 0.33 (0.10)*** [0.14; 0.52] |
| PrevOP-MMT high-impact exercise condition (vs. PrevOP-MMT active control condition) |  | -0.16 (1.40) [-2.90; 2.58] |
| PrevOP-MMT low-impact exercise condition (vs. PrevOP-MMT active control condition) |  | -0.86 (1.42) [-3.64; 1.91] |
| Sex |  | -0.42 (1.18) [-2.74; 1.89] |
| Age |  | -0.12 (0.08) [-0.27; 0.04] |
| Body mass index |  | 0.21 (0.12)^†^ [-0.03; 0.45] |
| Divorced |  | -0.44 (1.54) [-3.47; 2.58] |
| VAS-pain |  | 1.03 (0.40)* [0.24; 1.81] |
| Positive outcome expectancies |  | -0.48 (0.65) [-1.76; 0.80] |
| Source of self-efficacy: negative affect |  | -0.11 (0.55) [-1.19; 0.98] |
| **DV: WOMAC-stiffness at M24** |  |  |
| PrevOP-PAP intervention condition  (vs. PrevOP-PAP active control condition) | -0.28 (0.56) [-1.39; 0.82] | -0.09 (0.55) [-1.15; 0.98] |
| WOMAC-stiffness at M0 | 0.33 (0.06)*** [0.20; 0.45] | 0.23 (0.07)*** [0.10; 0.37] |
| PrevOP-MMT high-impact exercise condition (vs. PrevOP-MMT active control condition) |  | 0.35 (0.67) [-0.95; 1.66] |
| PrevOP-MMT low-impact exercise condition (vs. PrevOP-MMT active control condition) |  | -0.39 (0.68) [-1.71; 0.94] |
| Sex |  | -0.17 (0.57) [-1.29; 0.95] |
| Age |  | -0.04 (0.04) [-0.12; 0.04] |
| Body mass index |  | 0.14 (0.06)* [0.02; 0.25] |
| Divorced |  | 0.11 (0.74) [-1.35; 1.57] |
| VAS-pain |  | 0.39 (0.16)* [0.07; 0.71] |
| Positive outcome expectancies |  | 0.19 (0.31) [-0.42; 0.81] |
| Source of self-efficacy: negative affect |  | -0.05 (0.27) [-0.57; 0.46] |

*Note.* *N* = 241 participants. Coefficients are unstandardized. Dichotomous covariates (coded 1/0): PrevOP-PAP intervention condition (coded 1, vs. PrevOP-PAP active control condition, coded 0), PrevOP-MMT high-impact exercise condition (coded 1, vs. PrevOP-MMT active control condition, coded 0), PrevOP-MMT low-impact exercise condition (coded 1, vs. PrevOP-MMT active control condition, coded 0), sex male (coded 1, vs. female, coded 0), divorced (coded 1, vs. not divorced, coded 0). Continuous covariates (grand-mean centered, per one point increase): OAK symptoms (WOMAC) at M0, MVPA at M0, age, body mass index, VAS-pain, positive outcome expectancies, source of self-efficacy: negative affect. Est. = Estimate; *SE* = Standard error; CI = Confidence interval; OAK = Osteoarthritis of the knee; M0 = Baseline; M12 = 12-months follow-up; M24 = 24-months follow-up; PrevOP-MMT = PrevOP-Main Medical Trial; PrevOP-PAP = PevOP-Psychological Adherence Program; VAS = visual analogue scale; WOMAC = Western Ontario and McMaster Universities Osteoarthritis Index [4].

† *p* < .10; * *p* < .05; ** *p* < .01; *** *p* < .001

**Additional Table 2**

*Manifest Path Models Predicting Participants‘ Functional Limitations Associated with Osteoarthritis of the Knee*

|  | **Model 1**  **Dependent variable: WOMAC-functional limitations at M24** | | | **Model 2**  **Dependent variable: WOMAC-functional limitations at M24** | | |
| --- | --- | --- | --- | --- | --- | --- |
|  | **Direct Effect** | | **Indirect Effect** | **Direct Effect** | | **Indirect Effect** |
|  | MVPA at M12 | WOMAC-functional limitations at M24 | EV → M → DV | MVPA at M12 | WOMAC-functional limitations at M24 | EV → M → DV |
|  | Est (*SE*)  95% CI | Est (*SE*)  95% CI | Est (*SE*)  95% CI | Est (*SE*)  95% CI | Est (*SE*)  95% CI | Est (*SE*)  95% CI |
| PrevOP-PAP intervention condition  (vs. PrevOP-PAP active control condition) | -3.90 (3.34) [-10.47; 2.33] | -6.56 (3.28)* [-12.95; -0.34] | 0.19 (0.59) [-0.45; 2.30] | -3.37 (3.04) [-9.33; 2.60] | -5.77 (3.26)^†^ [-12.16; 0.61] | 0.09 (0.35) [-0.61; 0.78] |
| **Mediator:** |  |  |  |  |  |  |
| MVPA at M12 |  | -0.05 (0.11) [-0.25; 0.17] |  |  | -0.03 (0.10) [-0.23; 0.18] |  |
| **Covariates:** |  |  |  |  |  |  |
| WOMAC-functional limitations at M0 | -0.10 (0.06)^†^ [-0.22; 0.01] | 0.45 (0.08)*** [0.29; 0.60] | 0.00 (0.01) [-0.01; 0.05] | -0.09 (0.07) [-0.23; 0.06] | 0.35 (0.08)*** [0.20; 0.50] | 0.00 (0.01) [-0.02; 0.02] |
| MVPA at M0 | 0.72 (0.07)*** [0.57; 0.86] | 0.02 (0.12) [-0.21; 0.24] | -0.03 (0.08) [-0.18; 0.12] | 0.71 (0.06)*** [0.60; 0.82] | 0.01 (0.10) [-0.18; 0.20] | -0.02 (0.07) [-0.16; 0.12] |
| PrevOP-MMT high-impact exercise condition (vs. PrevOP-MMT active control condition) |  |  |  | 0.12 (3.73) [-7.20; 7.43] | 0.12 (3.96) [-7.64; 7.87] | -0.00 (0.09) [-0.19; 0.18] |
| PrevOP-MMT low-impact exercise condition (vs. PrevOP-MMT active control condition) |  |  |  | 0.28 (3.70) [-6.97; 7.52] | -0.02 (4.02) [-7.89; 7.86] | -0.01 (0.10) [-0.20; 0.19] |
| Sex |  |  |  | 0.78 (3.12) [-5.32; 6.89] | 0.57 (3.35) [-5.99; 7.14] | -0.02 (0.12) [-0.25; 0.21] |
| Age |  |  |  | -0.24 (0.21) [-0.65; 0.18] | 0.03 (0.24) [-0.44; 0.49] | 0.01 (0.02) [-0.04; 0.05] |
| Body mass index |  |  |  | -0.43 (0.35) [-1.11; 0.26] | 0.99 (0.36)** [0.29; 1.70] | 0.01 (0.05) [-0.08; 0.10] |
| Divorced |  |  |  | 7.98 (3.95)* [0.24; 15.72] | -5.41 (4.44) [-14.11; 3.29] | -0.20 (0.83) [-1.83; 1.43] |
| VAS-pain |  |  |  | 0.30 (0.99) [-1.64; 2.23] | 1.80 (1.04)^†^ [-0.24; 3.83] | -0.01 (0.04) [-0.09; 0.07] |
| Positive outcome expectancies |  |  |  | -2.10 (1.75) [-5.53; 1.32] | -0.18 (1.89) [-3.89; 3.53] | 0.05 (0.22) [-0.38; 0.49] |
| Source of self-efficacy: negative affect |  |  |  | 0.60 (1.49) [-2.33; 3.53] | -1.37 (1.62) [-4.56; 1.81] | -0.02 (0.07) [-0.16; 0.13] |
| ***R*^2^** | MVPA at M12: *R*^2^ = 0.57; OAK functional limitations at M24: *R*^2^ = 0.27 | | | MVPA at M12: *R*^2^ = 0.60; OAK functional limitations at M24: *R*^2^ = 0.33 | | |

*Note. N* = 241 participants. Because manifest path-models were fully saturated no model fit could be determined. Unstandardized coefficients. Dichotomous covariates (coded 1/0): PrevOP-PAP intervention condition (coded 1, vs. PrevOP-PAP active control condition), PrevOP-MMT high-impact exercise condition (coded 1, vs. PrevOP-MMT active control condition, coded 0), PrevOP-MMT low-impact exercise condition (coded 1, vs. PrevOP-MMT active control condition, coded 0), sex male (coded 1, vs. female, coded 0), divorced (coded 1, vs. not divorced, coded 0). Continuous covariates (grand-mean centered, per one point increase): WOMAC-functional limitations at M0, MVPA at M0, age, body mass index, VAS-pain, positive outcome expectancies, source of self-efficacy: negative affect. Est. = Estimate; *SE* = Standard error; CI = Confidence interval; EV = Exogenous variable; M = Mediator; DV = Dependent variable; MVPA = Moderate to Vigorous Physical Activity; M0 = Baseline; M12 = 12-months follow-up; M24 = 24-months follow-up; PrevOP-PAP = PevOP-Psychological Adherence Program; PrevOP-MMT = PrevOP-Main Medical Trial; VAS = visual analogue scale; WOMAC = Western Ontario and McMaster Universities Osteoarthritis Index [4]. † *p* < .10; * *p* < .05; ** *p* < .01; *** *p* < .001

**Additional Table 3**

*Manifest Path Models Predicting Participants‘ Pain Associated with Osteoarthritis of the Knee*

|  | **Model 1**  **Dependent variable: WOMAC-pain at M24** | | | **Model 2**  **Dependent variable: WOMAC-pain at M24** | | |
| --- | --- | --- | --- | --- | --- | --- |
|  | **Direct Effect** | | **Indirect Effect** | **Direct Effect** | | **Indirect Effect** |
|  | MVPA at M12 | WOMAC-pain at M24 | EV → M → DV | MVPA at M12 | WOMAC-pain at M24 | EV → M → DV |
|  | Est (*SE*)  95% CI | Est (*SE*)  95% CI | Est (*SE*)  95% CI | Est (*SE*)  95% CI | Est (*SE*)  95% CI | Est (*SE*)  95% CI |
| PrevOP-PAP intervention condition  (vs. PrevOP-PAP active control condition) | -3.37 (3.32) [-10.22; 3.09] | -2.99 (1.18)* [-5.36; -0.63] | 0.20 (0.26) [-0.08; 1.06] | -3.04 (3.06) [-9.05; 2.96] | -2.49 (1.14)* [-4.72; -0.26] | 0.19 (0.22) [-0.23; 0.61] |
| **Mediator:** |  |  |  |  |  |  |
| MVPA at M12 |  | -0.06 (0.03)^†^ [-0.12; 0.00] |  |  | -0.06 (0.03)^†^ [-0.13; 0.00] |  |
| **Covariates:** |  |  |  |  |  |  |
| WOMAC-pain at M0 | -0.12 (0.23) [-0.65; 0.29] | 0.52 (0.10)*** [0.34; 0.71] | 0.01 (0.02) [-0.01; 0.07] | -0.03 (0.26) [-0.55; 0.49] | 0.32 (0.10)*** [0.14; 0.51] | 0.00 (0.02) [-0.03; 0.03] |
| MVPA at M0 | 0.73 (0.07)*** [0.57; 0.86] | 0.04 (0.03) [-0.03; 0.11] | -0.04 (0.02)^†^ [-0.09; 0.00] | 0.71 (0.06)*** [0.60; 0.82] | 0.03 (0.03) [-0.04; 0.09] | -0.05 (0.03)^†^ [-0.09; 0.00] |
| PrevOP-MMT high-impact exercise condition (vs. PrevOP-MMT active control condition) |  |  |  | -0.20 (3.74) [-7.53; 7.13] | -0.19 (1.38) [-2.90; 2.52] | 0.01 (0.24) [-0.45; 0.48] |
| PrevOP-MMT low-impact exercise condition (vs. PrevOP-MMT active control condition) |  |  |  | 0.38 (3.71) [-6.89; 7.66] | -0.93 (1.41) [-3.68; 1.83] | -0.02 (0.23) [-0.48; 0.44] |
| Sex |  |  |  | 0.94 (3.13) [-5.20; 7.07] | -0.31 (1.17) [-2.60; 1.98] | -0.06 (0.20) [-0.45; 0.34] |
| Age |  |  |  | -0.26 (0.21) [-0.67; 0.16] | -0.15 (0.08)^†^ [-0.31; 0.01] | 0.02 (0.02) [-0.01; 0.05] |
| Body mass index |  |  |  | -0.58 (0.34)^†^ [-1.24; 0.08] | 0.15 (0.12) [-0.10; 0.39] | 0.04 (0.03) [-0.02; 0.10] |
| Divorced |  |  |  | 8.11 (4.01)* [0.24; 15.98] | 0.03 (1.55) [-3.01; 3.07] | -0.51 (0.38) [-1.26; 0.23] |
| VAS-pain |  |  |  | -0.12 (1.06) [-2.19; 1.95] | 1.06 (0.40)** [0.28; 1.85] | 0.01 (0.07) [-0.12; 0.14] |
| Positive outcome expectancies |  |  |  | -2.11 (1.75) [-5.54; 1.32] | -0.55 (0.66) [-1.84; 0.74] | 0.13 (0.13) [-0.13; 0.40] |
| Source of self-efficacy: negative affect |  |  |  | 0.49 (1.49) [-2.44; 3.42] | 0.00 (0.55) [-1.09; 1.09] | -0.03 (0.10) [-0.22; 0.16] |
| ***R*^2^** | MVPA at M12: *R*^2^ = 0.56; OAK pain at M24: *R*^2^ = 0.28 | | | MVPA at M12: *R*^2^ = 0.60; OAK pain at M24: *R*^2^ = 0.35 | | |

*Note. N* = 241 participants. Because manifest path-models were fully saturated no model fit could be determined. Unstandardized coefficients. Dichotomous covariates (coded 1/0): PrevOP-PAP intervention condition (coded 1, vs. PrevOP-PAP active control condition, coded 0), PrevOP-MMT high-impact exercise condition (vs. PrevOP-MMT active control condition), PrevOP-MMT low-impact exercise condition (coded 1, vs. PrevOP-MMT active control condition, coded 0), sex male (coded 1, vs. female, coded 0), divorced (coded 1, vs. not divorced, coded 0). Continuous covariates (grand-mean centered, per one point increase): WOMAC-pain at M0, MVPA at M0, age, body mass index, VAS-pain, positive outcome expectancies, source of self-efficacy: negative affect. Est. = Estimate; *SE* = Standard error; CI = Confidence interval; EV = Exogenous variable; M = Mediator; DV = Dependent variable; MVPA = Moderate to Vigorous Physical Activity; M0 = Baseline; M12 = 12-months follow-up; M24 = 24-months follow-up; PrevOP-PAP = PevOP-Psychological Adherence Program; PrevOP-MMT = PrevOP-Main Medical Trial; VAS = visual analogue scale; WOMAC = Western Ontario and McMaster Universities Osteoarthritis Index [4]. † *p* < .10; * *p* < .05; ** *p* < .01; *** *p* < .001

**Additional Table 4**

*Manifest Path Models Predicting Participants‘ Stiffness Associated with Osteoarthritis of the Knee*

|  | **Model 1**  **Dependent variable: WOMAC-stiffness at M24** | | | **Model 2**  **Dependent variable: WOMAC-stiffness at M24** | | |
| --- | --- | --- | --- | --- | --- | --- |
|  | **Direct Effect** | | **Indirect Effect** | **Direct Effect** | | **Indirect Effect** |
|  | MVPA at M12 | WOMAC-stiffness at M24 | EV → M → DV | MVPA at M12 | WOMAC-stiffness at M24 | EV → M → DV |
|  | Est (*SE*)  95% CI | Est (*SE*)  95% CI | Est (*SE*)  95% CI | Est (*SE*)  95% CI | Est (*SE*)  95% CI | Est (*SE*)  95% CI |
| PrevOP-PAP intervention condition  (vs. PrevOP-PAP active control condition) | -3.38 (3.35) [-10.37; 2.85] | -0.30 (0.58) [-1.38; 0.89] | 0.02 (0.09) [-0.08; 0.35] | -3.39 (3.04) [-9.35; 2.58] | -0.10 (0.55) [-1.18; 0.97] | 0.00 (0.05) [-0.10; 0.11] |
| **Mediator:** |  |  |  |  |  |  |
| MVPA at M12 |  | -0.01 (0.02) [-0.04; 0.03] |  |  | -0.00 (0.02) [-0.03; 0.03] |  |
| **Covariates:** |  |  |  |  |  |  |
| WOMAC-stiffness at M0 | 0.15 (0.27) [-0.39; 0.68] | 0.33 (0.07)*** [0.18; 0.47] | 0.00 (0.01) [-0.02; 0.01] | 0.40 (0.39) [-0.35; 1.16] | 0.23 (0.07)*** [0.10; 0.37] | -0.00 (0.01) [-0.01; 0.01] |
| MVPA at M0 | 0.72 (0.08)*** [0.56; 0.86] | 0.01 (0.02) [-0.03; 0.04] | 0.00 (0.01) [-0.03; 0.02] | 0.71 (0.06)*** [0.60; 0.82] | -0.00 (0.02) [-0.03; 0.03] | -0.00 (0.01) [-0.02; 0.02] |
| PrevOP-MMT high-impact exercise condition (vs. PrevOP-MMT active control condition) |  |  |  | -0.06 (3.73) [-7.38; 7.26] | 0.35 (0.67) [-0.96; 1.66] | 0.00 (0.01) [-0.01; 0.01] |
| PrevOP-MMT low-impact exercise condition (vs. PrevOP-MMT active control condition) |  |  |  | -0.07 (3.71) [-7.35; 7.21] | -0.41 (0.68) [-1.74; 0.92] | 0.00 (0.01) [-0.01; 0.01] |
| Sex |  |  |  | 0.16 (3.17) [-6.04; 6.37] | -0.16 (0.57) [-1.29; 0.96] | -0.00 (0.01) [-0.01; 0.01] |
| Age |  |  |  | -0.25 (0.21) [-0.67; 0.16] | -0.04 (0.04) [-0.12; 0.03] | 0.00 (0.00) [-0.01; 0.01] |
| Body mass index |  |  |  | -0.61 (0.34)^†^ [-1.28; 0.05] | 0.13 (0.06)* [0.01; 0.25] | 0.00 (0.01) [-0.02; 0.02] |
| Divorced |  |  |  | 7.59 (3.98)^†^ [-0.21; 15.39] | 0.11 (0.75) [-1.37; 1.58] | -0.01 (0.12) [-0.25; 0.23] |
| VAS-pain |  |  |  | -0.76 (0.93) [-2.58; 1.07] | 0.40 (0.16)*  [0.07; 0.72] | 0.00 (0.01) [-0.02; 0.02] |
| Positive outcome expectancies |  |  |  | -2.37 (1.78) [-5.87; 1.13] | 0.20 (0.32) [-0.43; 0.83] | 0.00 (0.04) [-0.07; 0.08] |
| Source of self-efficacy: negative affect |  |  |  | 0.43 (1.49) [-2.49; 3.35] | -0.04 (0.27) [-0.57; 0.49] | -0.00 (0.01) [-0.01; 0.01] |
| ***R*^2^** | MVPA at M12: *R*^2^ = 0.56; OAK stiffness at M24: *R*^2^ = 0.14 | | | MVPA at M12: *R*^2^ = 0.60; OAK stiffness at M24: *R*^2^ = 0.25 | | |

*Note. N* = 241 participants. Because manifest path-models were fully saturated no model fit could be determined. Unstandardized coefficients. Dichotomous covariates (coded 1/0): PrevOP-PAP intervention condition (coded 1, vs. PrevOP-PAP active control condition, coded 0), PrevOP-MMT high-impact exercise condition (coded 1, vs. PrevOP-MMT active control condition, coded 0), PrevOP-MMT low-impact exercise condition (coded 1, vs. PrevOP-MMT active control condition, coded 0), sex male (coded 1, vs. female, coded 0), divorced (coded 1, vs. not divorced, coded 0). Continuous covariates (grand-mean centered, per one point increase): WOMAC-stiffness at M0, MVPA at M0, age, body mass index, VAS-pain, positive outcome expectancies, source of self-efficacy: negative affect. Est. = Estimate; *SE* = Standard error; CI = Confidence interval; EV = Exogenous variable; M = Mediator; DV = Dependent variable; MVPA = Moderate to Vigorous Physical Activity; M0 = Baseline; M12 = 12-months follow-up; M24 = 24-months follow-up; PrevOP-PAP = PevOP-Psychological Adherence Program; PrevOP-MMT = PrevOP-Main Medical Trial; VAS = visual analogue scale; WOMAC = Western Ontario and McMaster Universities Osteoarthritis Index [4].

† *p* < .10; * *p* < .05; ** *p* < .01; *** *p* < .001

**References**

1. Kellgren JH, Lawrence JS. Radiological assessment of osteo-arthrosis. Ann Rheum Dis. 1957;16(4):494–502.

2. Altman R, Asch E, Bloch D, Bole G, Borenstein D, Brandt K, et al. Development of criteria for the classification and reporting of osteoarthritis: classification of osteoarthritis of the knee. Arthritis Rheum. 1986;29(8):1039-49.

3. Knoll N, Hohl DH, Motter S, Keller J, Lange D, Felsenberg D, et al. Facilitating physical activity and reducing symptoms in patients with knee osteoarthritis: study protocol of a randomized controlled trial to test a theory-based PrevOP-psychological adherence program (PrevOP-PAP). BMC Musculoskelet Disord. 2018;19:221.

4. Bellamy N. WOMAC Osteoarthritis Index User Guide, Version V. Brisbane 2002.
